# Delayed diagnosis and associated factors among new pulmonary tuberculosis patients diagnosed at the emergency department of a tertiary care hospital in Porto Alegre, South Brazil: a prospective patient recruitment study

**DOI:** 10.1186/1471-2334-13-538

**Published:** 2013-11-13

**Authors:** Gracieli Nadalon Deponti, Denise Rossato Silva, Ana Cláudia Coelho, Alice Mânica Muller, Paulo de Tarso Roth Dalcin

**Affiliations:** 1 Universidade Federal do Rio Grande do Sul (UFRGS), Programa de Pós-Graduação em Ciências Pneumológicas da UFRGS, Serviço de Pneumologia, Hospital de Clínicas de Porto Alegre (HCPA), Porto Alegre, Brazil

**Keywords:** Tuberculosis, Risk factors, Patient delay, Health care system delay, Diagnosis

## Abstract

**Background:**

Control of tuberculosis (TB) depends on early diagnosis and treatment at the primary health care level. However, many patients are still diagnosed late with TB at hospitals. The present study aimed to investigate the delay in diagnosis of TB patients at the emergency department.

**Methods:**

This was a prospective study in a general, tertiary care, university-affiliated hospital of a city with a high prevalence of TB in Brazil. New TB patients ≥ 14 years diagnosed with pulmonary TB at the emergency department of Hospital de Clínicas de Porto Alegre were prospectively recruited between February 2010 and January 2012. The consenting patients meeting our inclusion criteria were interviewed using a pre-tested questionnaire. We evaluated the delay in time until diagnosis and identified factors associated with delayed diagnosis (patient and health care system delays).

**Results:**

We included 153 patients. The median total time of delay, patient delay, and health care system delay were 60 (interquartile range [IQR]: 30–90.5 days), 30 (lQR: 7–60 days), and 18 (IQR: 9–39.5 days) days, respectively. The factors that were independently associated with patient delay (time ≥ 30 days) were crack (odds ratio [OR] = 4.88, p = 0.043) and cocaine (OR = 6.68, p = 0.011) use. The factors that were independently associated with health care system delay (time ≥ 18 days) were weight loss (OR = 2.76, p = 0.025), miliary pattern (OR = 5.33, p = 0.032), and fibrotic changes (OR = 0.12, p = 0.013) on chest X-ray.

**Conclusions:**

Patient delay appears to be the main problem in this city with a high prevalence of TB in Brazil. The main factor associated with patient delay is drug abuse (crack and cocaine). Our study shows substance abuse programs need to be aware of control of TB, with health interventions focusing on TB education programs.

## Background

Tuberculosis (TB) is an infectious disease caused by *Mycobacterium tuberculosis* and remains a public health problem worldwide. Approximately 2 billion people, representing one third of the world’s population, are infected with TB. Eight million people develop active disease each year, resulting in 2 million deaths from TB annually. Brazil is ranked 17^th^ among 22 countries, and has the highest reported incidence of TB, with 42 cases/100,000 inhabitants in 2011 [1]. The city of Porto Alegre in southern Brazil had a rate of incidence of TB in 104 cases/100,000 inhabitants/year in 2011 [2].

Control of TB in the community depends on early diagnosis and treatment. In regions with a high prevalence of TB, an early diagnosis is considered as one performed between 2 to 3 weeks after the onset of clinical symptoms and a late diagnosis is that performed 4 weeks after this onset [3]. Therefore, an acceptable time delay in diagnosis of 3 or 4 weeks would be effective for disease control [4]. The total diagnosis delay is the sum of patient delay, and health system delay. The reported ranges of the average (median or mean) total diagnosis delay, patient delay, and health system delay are 25–185 days, 4.9–162 days, and 2–87 days, respectively, for low, middle, and high income countries [5].

The reasons for delay in diagnosis reflect factors related to the patient and the health care system. Factors related to the health system vary according to each country or region [6]. The factors related to the individual involve personal characteristics, and socioeconomic and demographic factors [6,7]. The main factors associated with diagnostic delay include human immunodeficiency virus (HIV) infection, a negative sputum smear, extrapulmonary TB, old age, female sex, alcoholism and substance abuse, low education level, coexistence of chronic cough and/or other lung diseases, rural residence, low access, low awareness of TB, among others [6]. In studies conducted in Brazil, female sex, cough, unemployment, and the inability to recognize symptoms as indicators of disease are factors independently associated with patient delay [8,9]. In addition, only female sex is independently associated with health care system delay [8]. Furthermore, unemployment, having given up smoking, and having lost weight are associated with total delay (patient and health care system delays) [10].

Understanding the factors related to delay in diagnosis of disease is essential to reduce the period of transmission, reduce the risk of exposure of other community members, and thus facilitate disease control. Few studies have evaluated these factors in tertiary care hospitals in regions with a high incidence of TB and HIV [6,11]. The objective of this study was to investigate the delay in diagnosis of TB patients at the emergency department (ED) of a tertiary care hospital, and to analyze factors associated with patient and health care system delays.

## Methods

### Study design and setting

We conducted a prospective study at the ED of the Hospital de Clinicas de Porto Alegre (HCPA). The HCPA is a tertiary hospital and a reference center for HIV patients in South Brazil. Porto Alegre is among the Brazilian cities with the highest incidence of TB, with 2123 cases registered in 2011, 200 deaths in patients co-infected with TB-HIV, and 50 deaths in patients who were HIV negative [2]. Furthermore, Porto Alegre has a TB-HIV co-infection rate of 35% [12]. In Brazil, the public health system provides TB health services and treatment free of charge at hospitals and health centers.

### Participants

The study population consisted of patients diagnosed with TB at the ED. Patients aged more than 14 years, diagnosed as new cases of pulmonary TB (with or without extrapulmonary TB), according to consensus criteria [13], were included in the study. The study was approved by the Research Ethics Committee of the HCPA (protocol 09–575). All patients or their legal guardians gave written informed consent to participate.

Pulmonary TB was diagnosed according to any of the following criteria established by the Brazilian Guidelines for Tuberculosis [13]: 1) detection by a direct test (Ziehl-Neelsen [ZN] method) with two positive samples; 2) detection by a direct test (ZN method) with one positive sample and a positive culture result for *M. tuberculosis* (in Löwenstein-Jensen [LJ] medium); 3) detection by a direct test (ZN method) with one positive sample and radiological findings compatible with TB; 4) only a positive culture result for *M. tuberculosis* (in LJ medium); or 5) the presence of clinical, epidemiological, and radiographic findings compatible with TB. Patients who failed to respond to questionnaires and those who refused to sign the consent form were excluded from the study.

### Data collection

For data collection, we used a standardized and pre-tested questionnaire with open and closed questions. The interviews were conducted by three interviewers who were previously trained at the time of admission. Factors that could be associated with delay in seeking care at health services, and delay in diagnosis and initiation of treatment for TB were collected. These factors included demographic characteristics, such as age (years), sex (male/female), ethnicity (white/non-white race), income (monthly household income), and education level (years of schooling). Behavioral characteristics included (1) smoking status as follows. Respondents who reported smoking at least 100 cigarettes in their lifetime and who, at the time of survey, smoked either every day or some days were defined as current smokers. Respondents who reported smoking at least 100 cigarettes in their lifetime and who, at the time of the survey, did not smoke at all were defined as former smokers. Respondents who reported never having smoked 100 cigarettes were defined as never smokers. (2) Alcohol use was investigated where alcohol abuse was defined as daily consumption of at least 30 g of alcohol for men and 24 g for women. (3) Drug use was defined as any use in the past 6 months. (4) Self-medication use was recorded. Clinical features of the disease included the clinical form of TB (pulmonary or pleuropulmonary), symptoms (cough, sputum production, night sweats, weight loss, fever, dyspnea, hemoptysis, chest pain, and fatigue), time since the first symptom, HIV status (patients were tested for HIV at the discretion of the ER physician), non-HIV immunosuppression (chronic renal failure, malignant tumors, transplant recipients, and those using prednisone or an equivalent [at a dose ≥ 15 mg/day for 4 weeks]), immunosuppressants (for 4 weeks or longer) or biological modifiers, such as etanercept and infliximab. Services sought before the ED (if yes or no, and the number of services sought), and the history of previous TB (and if the patient completed at least 6 months of treatment) were also collected. The diagnostic method (spontaneous sputum, induced sputum, and bronchoalveolar lavage) and chest X-ray results were obtained from the electronic medical records.

Patients were asked to estimate the time in days from onset of symptoms to the first clinical care (any clinicians, not only in the ED). The total time of delay was defined as the period from the beginning of any symptoms until the use of at least three antituberculosis drugs. Patient delay was defined as the period from the beginning of any symptoms of TB until the first medical care. Health system delay was defined as the period from the first medical care until the beginning of at least three antituberculosis drugs.

### Sample size calculation and statistical analysis

Calculation of sample size was performed based on a study by Ward et al. [14], where the variable that was significantly associated with delayed initiation of treatment in the health system was an age greater than 45 years (the prevalence of an age > 45 years was 59%). A previous study in the HCPA showed that the prevalence of an age greater than 45 years in patients with TB was 60% [15]. Therefore, considering a difference between the proportions in groups (patient delay [≥ 30 days versus < 30 days] and health system delay [≥ 18 days versus < 18 days]) of 25%, with the lower proportion of 40%, α = 0.05, and β = 20%, the total number of patients needed to be studied was 69 in each group.

The median patient delay and health care system delay were used to dichotomize the sample for analysis. Patients were evaluated according to patient delay (group 1: ≥ 30 days versus group 2: < 30 days) and health system delay (group 1: ≥ 18 days versus group 2: < 18 days). Data analysis was performed using SPSS 18.0 (Statistical Package for the Social Sciences, Chicago, IL). Data are shown as the number of cases, mean ± standard deviation (SD), or median with interquartile range (IQR). Categorical comparisons were performed by the chi-square test using Yates’s correction if indicated or by Fisher’s exact test. Continuous variables were compared using the *t*-test or Wilcoxon test. Multivariate logistic regression analysis was used to evaluate risk factors for patient and health care system delays. Multivariate logistic regression modeling attempted to use all factors associated (p < 0.10) with the outcome in univariate models. Stepwise logistic regression, with entry criteria of p < 0.10 and removal criteria of p > 0.05, was used to establish the final multivariate predictive model. This type of variable selection with bivariable selection is frequently used because nonsignificant risk factors in bivariable analysis may actually be significant risk factors in multivariable analysis. If the statistical p value of a risk factor in bivariable analysis is greater than an arbitrary value (often p = 0.1), then this factor will be allowed to compete for inclusion in multivariable analysis. Hierarchical logistic regression models with predictors added one at a time were also examined to evaluate possible collinearity among the predictors. The predictors selected in the final model were based on numerical and clinical significance. The goodness of fit of the multiple logistic regression models was assessed using the Hosmer–Lemeshow test. Odds ratios (ORs) and nominal 95% confidence intervals (CIs) are presented. A two-sided p value < 0.05 was considered significant for all analyses.

## Results

### General results

From February 2010 to January 2012, 418 patients were eligible for the study. Among pulmonary TB patients, we excluded 248 patients who were already receiving treatment at admission, 12 patients who refused to participate, and five patients because TB diagnosis was not confirmed. Therefore, 153 patients were included in the study.

Table 1 shows the patients’ characteristics. The median total delay (from onset of symptoms to initiation of treatment) was 60 days. The median patient delay was 30 days and the median health care system delay was 18 days.

**Table 1:** Patients’ characteristics

**Characteristics**	**n = 153**
Age, years (mean ± SD)	42.6 ± 15.7
Male sex, n (%)	90 (58.8)
White race, n (%)	88 (57.5)
< 8 years schooling, n (%)	81 (52.9)
Literate, n (%)	141 (92.2)
Living alone, n (%)	30 (19.6)
Belief in a religion, n (%)	126 (82.4)
Monthly household income < 1 standard minimum wage*, n (%)	51 (33.3)
Living in Porto Alegre, n (%)	101 (66.0)
Institutionalization, n (%)	23 (15)
Smoking habit, n (%)	
Never smoker	55 (35.9)
Current smoker	42 (27.5)
Former smoker	56 (36.6)
Alcoholism, n (%)	53 (34.6)
Drug use, n (%)	50 (32.7)
Marijuana, n (%)	29 (19.0)
Cocaine use, n (%)	27 (17.6)
Crack use, n (%)	35 (22.9)
HIV positive, n (%)	77 (51.1)
Non-HIV immunosuppression, n (%)	22 (14.4)
Sought care elsewhere before hospitalization, n (%)	113 (73.9)
Forms of TB, n (%)	
Pulmonary TB	127 (83.0)
Milliary TB	19 (12.4)
Pleuropulmonary TB	7 (4.6)
Total time until diagnosis (days), median (IR)	60.0 (30.0-90.5)
Patient delay (days), median (IR)	30.0 (7.0-60.0)
Health care system delay (days), median (IR)	18.0 (9.0-39.5)

TB = Tuberculosis; SD = Standard deviation, n = Number of cases, HIV = Human immunodeficiency virus, IR = Interquartile range.

*Approximately U$ 312 (2013).

### Patient delay

Table 2 shows comparative analysis for patient delay. Compared with Group 2 (< 30 days for patient delay), Group 1 (≥ 30 days for patient delay) had a significantly higher proportion of cocaine use (27.8 versus 6.8%, p = 0.001) and crack use (31.6 versus 13.5%; p = 0.013). The proportion of patients who sought treatment at another facility before going to the ED was significantly higher in Group 1 than in Group 2 (82.3 versus 64.9%, p = 0.023). In binary logistic regression analysis for patient delay, the factors that were independently associated with a time delay ≥ 30 days were crack use (OR = 4.88, p = 0.043) and cocaine use (OR = 6.68, p = 0.011).

**Table 2:** Univariate analysis and logistic regression analysis for patient delay

**Characteristics**	**Group 1 (≥30 days)**	**Group 2 (<30 days)**		**Univariate analysis**			**Logistic regression**	
			**OR**	**95% CI**	**p**	**Adjusted OR**	**95% CI**	**p**
Age, years (mean ± SD)	44.8 ± 16.0	40.2 ± 15.0	0.98	0.96-1.01	0.069	1.03	0.99-1.05	0.064
Male sex, n (%)	51 (64.6)	39 (52.7)	1.28	0.92-1.78	0.185	1.02	0.48-2.20	0.960
White race, n (%)	42 (53.2)	46 (62.2)	0.84	0.62-1.14	0.336			
< 8 years schooling, n (%)	41 (51.9)	40 (54.1)	0.96	0.71-1.30	0.916			
Literate, n (% sim)	73 (92.4)	68 (91.9)	1.04	0.58-1.86	0.999			
Living alone, n (%)	17 (21.5)	13 (17.6)	1.12	0.79-1.61	0.681			
Belief in a religion, n (%)	65 (82.3)	61 (82.4)	1.00	0.66-1.49	0.999			
Monthly household income < 1 standard minimum wage*, n (%)	28 (35.4)	23 (31.1)	1.10	0.80-1.51	0.689			
Living in Porto Alegre, n (%)	49 (62.0)	52 (70.3)	0.84	0.62-1.14	0.365			
Institutionalization, n (%)	13 (16.5)	10 (13.5)	1.22	0.57-2.61	0.778			
Current or former smoker	56 (70.9)	42 (56.8)	1.35	0.99-1.87	0.099	0.72	0.34-1.55	0.397
Alcoholism, n (%)	27 (34.2)	26 (35.1)	0.98	0.71-1.36	1.000			
Drug use, n (%)	32 (40.5)	18 (24.3)	1.40	1.04-4.25	0.05	1.68	0.73-3.88	0.225
Marijuana use, n (%)	16 (20.3)	13 (17.6)	1.09	0.75-1.57	0.828			
Cocaine use, n (%)	22 (27.8)	5 (6.8)	1.80	1.38-2,34	0.001	6.68	1.54-28.9	0.011
Crack use, n (%)	25 (31.6)	10 (13.5)	1.56	1.17-2.08	0.013	4.88	1.05-22.74	0.043
Symptoms, n (%)								
Cough	70 (88.6)	63 (85.1)	1.17	0.70-1.95	0.692			
Night sweats	55 (69.6)	47 (63.5)	1.15	0.81-1.61	0.529			
Fever	65 (82.3)	60 (81.1)	1.04	0.69-1.56	0.999			
Sputum production	56 (70.9)	42 (56.8)	1.37	0.96-1.95	0.099			
Hemoptysis	16 (20.3)	9 (12.2)	1.30	0.93-1.83	0.257			
Weight loss	62 (78.5)	55 (74.3)	1.12	0.76-1.65	0.678			
Dyspnea	52 (65.8)	43 (58.1)	1.17	0.85-1.64	0.414			
Chest pain	41 (51.9)	29 (39.2)	1.28	0.94-1.74	0.157			
Fatigue	32 (40.5)	21 (28.4)	1.29	0.95-1.74	0.160			
HIV positive, n (%)	41 (64.1)	36 (58.1)	1.14	0.79-1.63	0.612			
Non-HIV immunosuppression, n (%)	12 (15.2)	10 (13.5)	1.07	0.70-1.62	0.948			
Sought care elsewhere before hospitalization, n (%)	65 (82.3)	48 (64.9)	1.64	1.05-2.58	0.023			
Previous TB, n (%)	16 (20.3)	8 (10.8)	1.37	0.98-1.91	0.167			
Completed treatment, n (%)	8 (50.0)	6 (75.0)	0.72	0.41-1.14	0.388			
Knows someone with TB, n (%)	41 (51.9)	36 (48.6)	1.07	0.78-1.45	0.810			

TB = Tuberculosis; SD = Standard deviation, n = Number of cases, HIV = Human immunodeficiency virus.

*Approximately U$ 312 (2013).

### Health care system delay

Table 3 shows comparative analysis for health system delay. The proportion of patients reporting sputum production was significantly higher in Group 2 (< 18 days for health care system delay) than in Group 1 (≥ 18 days for health care system delay) (73.3 versus 55.1%, p = 0.029), and the proportion of patients reporting previous TB (22.7 versus 9.0%, p = 0.035) and patients with fibrotic changes on chest X-ray (17.3 versus 2.6%, p = 0.005). In multivariate analysis, the factors that were independently associated with a time delay ≥ 18 days were weight loss (OR = 2.76, p = 0.025), chest radiography with a miliary pattern (OR = 5.33, p = 0.032), and fibrotic changes (OR = 0.12, p = 0.013).

**Table 3:** Univariate analysis and logistic regression for health care system delay

**Characteristics**	**Group 1 (≥18 days)**	**Group 2 (<18 days)**		**Univariate analysis**			**Logistic regression**	
			**OR**	**95% CI**	**p**	**Adjusted OR**	**95% CI**	**p**
Age, years (mean ± SD)	43.8 ± 15.83	41.28 ± 15.57	0.99	0.97-1.01	0.321	0.98	0.96-1.01	0.081
Male sex, n (%)	47 (60.3)	43 (57.1)	1.06	0.77-1.76	0.839	1.16	0.53-2.4	0.720
White race, n (%)	48 (61.5)	40 (53.3)	1.18	0.85-1.64	0.388			
Current or former smoker	50 (34.1)	48 (64.0)	1.01	0.72-1.38				
Alcoholism, n (%)	31 (39.7)	22 (29.3)	1.24	0.92-1.69	0.324			
Drug use, n (%)	26 (33.3)	24 (32.0)	1.03	0.74-1.43	0.997			
Marijuana use, n (%)	16 (20.5)	13 (17.3)	1.10	0.76-1.60	0.768			
Cocaine use, n (%)	17 (21.8)	10 (13.3)	1.30	0.93-1.83	0.246			
Crack use, n (%)	18 (23.1)	17 (22.7)	1.01	0,70-1.46	0.999			
Symptoms, n (%) Cough	66 (84.6)	67 (89.3)	0.83	0.56-1.23	0.532			
Sputum production	43 (55.1)	55 (73.3)	0.69	0.51-0.93	0.029	0.45	0.20-1.01	0.052
Hemoptysis	12 (15.4)	13 (17.3)	0.93	0.60-1.45	0.555			
Fever	63 (80.8)	62 (82.7)	0.94	0.64-1.38	0.925			
Weight loss	65 (83.3)	52 (69.3)	1.54	0.97-2.45	0.064	2.76	1.14-6.71	0.025
Dyspnea	49 (62.8)	46 (61.3)	1.03	0.75-1.43	0.982			
Night sweats	58 (74.4)	44 (58.7)	1.45	0.99-2.12	0.059	1.74	0.77-3.93	0.184
Chest pain	36 (46.2)	34 (45.3)	1.01	0.74-1.39	0.999			
Fatigue	29 (37.2)	23 (30.7)	1.18	0.86-1.61	0.408			
Had consultation at the first health service sought, n (%)	72 (92.3)	74 (98.7)	0.58	0.41-0.81	0.135			
Chest x-ray performed, n (%)	65 (90.3)	69 (93.2)	0.94	0.69-1.28	0.202			
Previous TB, n (%)	7 (9.0)	17 (22.7)	0.53	0.28-1.01	0.035	0.50	0.15-1.66	0.256
Completed treatment, n (%)	4 (57.1)	10 (58.8)	0.95	0.27-3.35	0.999			
HIV positive, n (%)	42 (60.9)	35 (61.4)	0.99	0.72-1.37	0.999			
Radiographic pattern, n (%)								
Cavitation	12 (15.6)	22 (29.3)	0.64	0.40-1.04	0.066	0.45	0.19-1.08	0.074
Pleural effusion	4 (5.1)	2 (2.7)	1.32	0.74-2.39	0.682			
Milliary pattern	11 (14.1)	3 (4.0)			0.059	5.33	1.15-24.56	0.032
Consolidation	41 (52.6)	40 (53.3)			0.999			
Fibrotic changes	2 (2.6)	13 (17.3)			0.005	0.12	0.02- 0.64	0.013
Reticular infiltrates	28 (35.9)	21 (28.0)			0.382			
Forms of TB								
Pulmonary TB	75 (96.2)	74 (98.7)			0.620			
Pleuropulmonary TB	3 (3.8)	1 (1.3)			0.640			
Pulmonary + milliary TB	12 (15.4)	7 (9.3)			0.374			

TB = Tuberculosis; SD = Standard deviation, n = Number of cases, HIV = Human immunodeficiency virus, OR = Odds ratio, 95% CI = 95% confidence interval.

## Discussion

This study evaluated patients diagnosed with TB in the ED of a university hospital, in a city with a high prevalence of this disease. The total time from symptom onset to the start of treatment was too long, and the delay attributed to the patient was greater than the delay attributed to the health system. The factors that were independently associated with patient delay were cocaine and crack use. Factors that were independently associated with health system delay were weight loss and miliary pattern on chest X-rays, while the presence of fibrotic changes on chest radiography was negatively associated with health system delay. We did not find any association between delayed diagnosis and some factors usually associated with delay in previous studies, such as HIV infection, negative sputum smear, old age, female sex, low educational level, poverty, and smoking [6].

To date, there is no consensus on an "acceptable" time for a delay in diagnosis of TB, but it depends on the epidemiology and health services of each country [14]. In underdeveloped or developing countries with highly endemic TB, the total time delay exceeds 120 days, while in many developing countries, the total time delay is approximately 50 days [6].

Brazil is considered a country with high endemic TB [1]. However, the total time delay until diagnosis of TB that we found (60 days) in the current study is unacceptably too long. A long time delay has also been reported by studies conducted in other regions of the country [8-10]. The southeast region reported total time delays of 68 days [8] and 77 days [9], and a study in the northeastern region reported a total time of 90 days [10]. These findings indicate that in Brazil, there is a need for greater awareness of the symptoms of TB. There is also a need for early diagnosis of TB to attempt to reduce this time to 3–4 weeks as recommended by international guidelines for the control of disease [3].

In a systematic review [5], the total time delay in diagnosis in countries with low and highly endemic TB was 25 and 187 days, the patient delay was 4.9 and 162 days, and the health system delay was 2 and 87 days, respectively. Our study found similar time delays to those reported in Brazilian studies [8,9]; the median patient delay ranged from 30 to 56 days and the median health system delay ranged from 14 to 21 days.

We found an association between patient delay and drug use, particularly cocaine and crack. Patients who abuse substances are more susceptible to diseases and have a greater period of transmissibility of the disease, because they do not seek health services early, or use TB drugs irregularly [16]. Furthermore, in accordance with our findings, a previous study showed that drug users had a delay in initiation of treatment compared with patients who did not use drugs [17].

Despite TB control programs’ recommendation that diagnosis should be made at the primary health care level, most patients are still diagnosed in hospitals, especially public hospitals [18,19]. In 2007, in Porto Alegre, 38.98% of TB cases were reported by hospitals [20]. In our study, most patients with a diagnostic delay of longer than 30 days sought other health services before a hospital. Previous studies have shown that a delay in diagnosis is closely related to poor access to health care, either by distance from health services or difficulties in performing exams, and prescription of medications other than antituberculosis drugs [6,10]. This may indicate the need for training professionals at primary health care clinics to recognize TB symptoms, allowing early diagnosis and treatment.

Another factor related to health system delay in our study was weight loss. Similar studies have shown that weight loss is associated with a delay in diagnosis and treatment initiation [10,21,22]. Another study showed that weight loss is an independent predictor for pulmonary TB diagnosis [23]. Furthermore, the presence of radiographic changes, such as a miliary pattern, is associated with delayed initiation of treatment [23,24]. However, there is the possibility that weight loss and a military pattern on chest X-ray are more likely to occur in someone who has had untreated TB for a long period, and these findings may in fact be a result of a delay in treatment. Fibrotic changes and previous TB were associated with a shorter time to initiation of treatment in our study. A history of previous TB has been reported as an independent predictor for pulmonary TB diagnosis, and fibrotic changes may be associated with some symptoms of TB, such as hemoptysis, which facilitates the diagnosis and prompt initiation of TB treatment [23].

Half of the patients included in our study were TB-HIV patients. The TB-HIV co-infection rate in the city of Porto Alegre is the highest in the country (35%) [25]. This high prevalence of TB-HIV co-infection is probably because the study hospital is a reference center for HIV patients in South Brazil. In addition, HIV infection was not significantly associated with delays in our study. HIV is known to delay TB diagnosis because of non-specific and atypical clinical presentation [26,27]. However, our findings are in agreement with other studies [28-30] that found no association between HIV positivity and delayed diagnosis. In a previous investigation, HIV-positive TB patients suffered more serious symptoms when TB was diagnosed, which prompted them to visit the hospital and increased the suspicion of TB by the clinician [30].

The main limitation of the present study is that we only used a sample of the population served by the hospital. A control group of individuals diagnosed and treated at primary care health clinics could be useful for identifying the factors related to a delay in diagnosis. Furthermore, the study was conducted in a single center and referral center in the public system, and probably included patients with more severe disease and lower socioeconomic status. These factors did not affect the results, but they are specifically applicable to populations similar to that in our study. In addition, we did not evaluate some factors related to health system delay, such as the type of provider (public, private, and complimentary), rural/urban residence, and availability and accessibility of services. Finally, there was the possibility of recall bias associated with the time in days from onset of symptoms to the first clinical care. Despite these concerns, our study still showed some interesting findings.

Although we found some delay in the health care system, patient delay appears to be more important. The main factor associated with patient delay was drug abuse (crack and cocaine). Drug users may have less access to routine medical care, potentially leading to delayed diagnosis. Moreover, in a study with marijuana users, patients reported that despite feeling ill and having increased coughing, they delayed seeking care until TB was advanced [31], resulting in an extended period of infectiousness [32,33]. In addition, another study demonstrated that patients who abuse substances are less likely to complete TB screening [34]. There is a need for public health interventions among drug users, focusing on TB education programs. The use of some successful strategies, such as needle exchange programs [35,36], should also be considered.

## Conclusions

In conclusion, this study shows that in patients with TB diagnosed in the ED, the total time from symptom onset to TB diagnosis is too long. Additionally, patient delay appears to be the main problem in delay in diagnosis of TB in our study setting. The main factor associated with patient delay is drug abuse (crack and cocaine). Control of TB and substance abuse programs need to be closely linked, and health interventions should focus on TB education programs.

## Competing interests

The authors declare that they have no competing interests.

## Authors’ contributions

GND participated in the design of the study, collected the data, and wrote the manuscript. DRS participated in the conception and design of the study, performed statistical analysis, and wrote the manuscript. ACC and AMM collected the data and helped to draft the manuscript. PTRD participated in the conception and design of the study, performed statistical analysis, and wrote the manuscript. All authors read and approved the final manuscript.

## Pre-publication history

The pre-publication history for this paper can be accessed here:

http://www.biomedcentral.com/1471-2334/13/538/prepub
